# Treatment of Enlarged Tonsils

**Published:** 1869-04

**Authors:** J. Mason Warren

**Affiliations:** Surgeon to the Massachusetts Hospital


TREATMENT OF ENLARGED TONSILS.—By J. MASON WARREN, M.D., Surgeon to the Massachusetts Hospital. (*Surgical Observations, with Cases and Observations*; and *Medical Times and Gazette*, October 3.)

Dr. Warren is an advocate for excision of tonsils, and states that he has performed this minor operation more than five hundred times with satisfactory results. In cases of enlarged tonsils with otorrhœa, deafness, difficulty of respiration, and other disturbances caused by obstruction to the free ingress of air, partial removal of the affected glands has given great relief. The following are his conclusions on the results of such treatment:

"From a review of a large number of cases, I find that many of the children were of a scrofulous constitution; that the enlargement of the tonsils caused great local trouble, attended with considerable constitutional disturbance; that the patient was much more liable to inflammatory attacks of the throat than in cases where this condition does not exist; and that they were less liable, after the operation, to these attacks.

"In about half of all the cases, and in about two-thirds of those of children, deformity of the chest existed. Whether this depended on the general constitutional habit of the patient, or was induced by the obstruction in the throat to the free passage of air, the accounts received as to the exact time when either affection was first observed were not sufficiently accurate to justify a decision. It is certain, however, that this deformity does not increase, but rather diminishes, after the removal of the obstruction in the throat. The operation is a simple one, attended with no danger, and almost always affords immediate relief to the symptoms."

The instrument generally used by Dr. Warren is a guillotine without any steel movable needle for piercing the tonsil, as this is thought to be a useless addition.

